# The First and Second Reports of the Medical Missionary Society in China; with Minutes of Proceedings, Hospital Reports, &c.

**Published:** 1842-07

**Authors:** 


Art. IV.—*The First and Second Reports of the Medical Missionary Society in China; with Minutes of Proceedings, Hospital Reports, &c.—Macao,* 1841. 8vo, pp. 68.

These Reports form a complete account of the Society's operations from the commencement, which are extremely creditable to all concerned. They show the activity with which the society carries on its operations, and strikingly indicate not merely the benefits likely to be derived from it eventually, as a means of civilization, but even the vast present and immediate good in healing the diseases of the people. No less than 6300 cases were treated by the medical officers, Mr. Hodson, Dr. Parker, Mr. Lockhart, and Mr. Hobson during the first three years. A good deal of curious information is given respecting the diseases of the Chinese, which we regret being unable to lay before our readers; but we earnestly recommend this excellent society to their attention and patronage.

